# Nonfunctional *coq10* mutants maintain the ERMES complex and reveal true phenotypes associated with the loss of the coenzyme Q chaperone protein Coq10

**DOI:** 10.1016/j.jbc.2024.107820

**Published:** 2024-09-27

**Authors:** Noelle Alexa Novales, Kelsey J. Feustel, Kevin L. He, Guillaume F. Chanfreau, Catherine F. Clarke

**Affiliations:** Department of Chemistry & Biochemistry, Molecular Biology Institute, UCLA, Los Angeles, California, USA

**Keywords:** coenzyme Q, Coq10, CoQ synthome, endoplasmic reticulum-mitochondria encounter structure, lipid, Mdm12, mitochondrial metabolism, *Saccharomyces cerevisiae*, START domain, ubiquinone

## Abstract

Coenzyme Q (CoQ) is a redox-active lipid molecule that acts as an electron carrier in the mitochondrial electron transport chain. In *Saccharomyces cerevisiae*, CoQ is synthesized in the mitochondrial matrix by a multisubunit protein–lipid complex termed the CoQ synthome, the spatial positioning of which is coordinated by the endoplasmic reticulum-mitochondria encounter structure (ERMES). The *MDM12* gene encoding the cytosolic subunit of ERMES is coexpressed with *COQ10*, which encodes the putative CoQ chaperone Coq10, *via* a shared bidirectional promoter. Deletion of *COQ10* results in respiratory deficiency, impaired CoQ biosynthesis, and reduced spatial coordination between ERMES and the CoQ synthome. While Coq10 protein content is maintained upon deletion of *MDM12*, we show that deletion of *COQ10* by replacement with a *HIS3* marker results in diminished Mdm12 protein content. Since deletion of individual ERMES subunits prevents ERMES formation, we asked whether some or all of the phenotypes associated with *COQ10* deletion result from ERMES dysfunction. To identify the phenotypes resulting solely due to the loss of Coq10, we constructed strains expressing a functionally impaired (*coq10-L96S*) or truncated (*coq10-R147∗*) Coq10 isoform using CRISPR-Cas9. We show that both *coq10* mutants preserve Mdm12 protein content and exhibit impaired respiratory capacity like the *coq10*Δ mutant, indicating that Coq10’s function is vital for respiration regardless of ERMES integrity. Moreover, the maintenance of CoQ synthome stability and efficient CoQ biosynthesis observed for the *coq10-R147∗* mutant suggests these deleterious phenotypes in the *coq10*Δ mutant result from ERMES disruption. Overall, this study clarifies the role of Coq10 in modulating CoQ biosynthesis.

Coenzyme Q (ubiquinone or CoQ) is an essential redox-active lipid molecule found in the plasma membranes and endomembranes of all eukaryotic species (1, 2). Proper localization of CoQ is dependent on its hydrophobic tail, which enables CoQ to anchor itself into the midplane of lipid bilayers and is comprised of a species-specific number of isoprene units (indicated by *n* in CoQ_n_) (3). The fully substituted benzoquinone head group imparts its characteristic redox activity, enabling CoQ to perform its most well-known function as an electron and proton carrier within the mitochondrial electron transport chain (1, 2). Other processes that rely on CoQ’s ability to act as electron acceptor include sulfide detoxification, proline catabolism, and choline degradation (1, 2). Additionally, the fully reduced form CoQH_2_ (ubiquinol) serves as a vital lipid-soluble antioxidant capable of ameliorating peroxidation of lipids in cellular membranes (1, 4).

In *Saccharomyces cerevisiae* (yeast), biosynthesis of CoQ_6_ (the CoQ isoform synthesized by yeast) requires 14 nuclear-encoded proteins: Coq1-Coq11, Yah1, Arh1, and Hfd1 (2, 5). Of the Coq polypeptides, Coq1 synthesizes hexaprenyldiphosphate, which is subsequently attached to the C3 position of the ring precursor 4-hydroxybenzoic acid or *para-*aminobenzoic acid (pABA), by Coq2 (2, 5). The remaining headgroup modifications are then carried out by several other Coq polypeptides to generate the final product, CoQ_6_/CoQ_6_H_2_ (Fig. 1*A*). Efficient CoQ_6_ biosynthesis requires many of the aforementioned Coq polypeptides (Coq3-Coq9 and Coq11) to localize to the matrix side of the inner mitochondrial membrane where they assemble into a high-molecular-weight complex termed the CoQ synthome (2, 5). Individual deletion of genes encoding Coq1-Coq9 results in abolished CoQ_6_ biosynthesis and an inability to respire, as these Coq polypeptides are required for catalytic steps within the CoQ biosynthetic pathway and/or structural stability of the CoQ synthome (6, 7).

**Figure 1:**
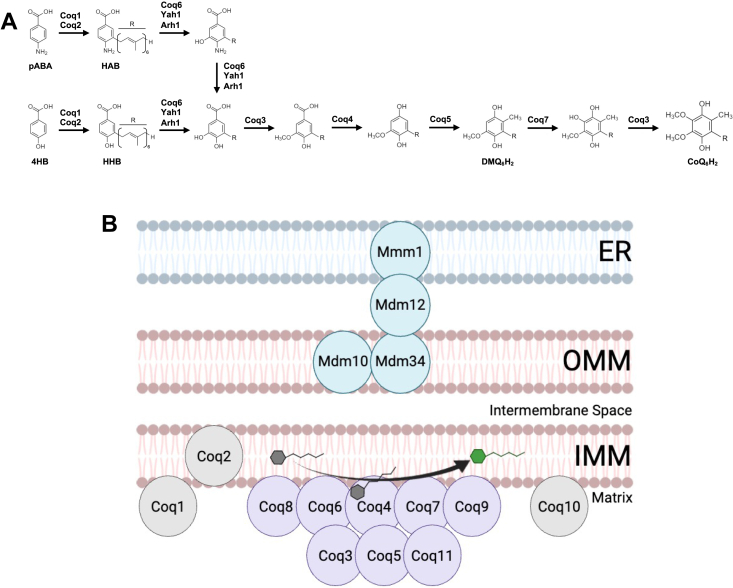
**CoQ biosynthesis in yeast requires the CoQ synthome to assemble adjacent to ERMES contact sites.***A*, the proposed CoQ biosynthetic pathway in *Saccharomyces cerevisiae*. *B*, schematic depicting the CoQ synthome positioned adjacent to the ERMES complex. CoQ synthome members are represented in purple and ERMES components are highlighted in *turquoise*. Coq1, Coq2, and Coq10 (*gray*) are not members of the CoQ synthome but are still required to observe efficient CoQ_6_ biosynthesis. This image was generated using BioRender.com. CoQ, coenzyme Q; ERMES, endoplasmic reticulum-mitochondria encounter structure.

Unlike most yeast *coq* mutants, the *COQ10* deletion (*coq10*Δ) mutant contains near WT amounts of CoQ_6_ in the stationary phase, yet still displays a respiratory-deficient growth phenotype, impaired *de novo* CoQ_6_ biosynthesis during log phase growth, and a destabilized CoQ synthome (8, 9, 10, 11). The NMR structure of the *Caulobacter crescentus* Coq10 ortholog, CC1736, revealed the presence of a steroidogenic acute regulatory protein-related lipid transfer (START) domain (12), shown to be capable of binding CoQ and its late-stage quinone intermediates *in vitro* (13). Additionally, Coq10 isolated from either *S. cerevisiae* or the fission yeast, *Schizosaccharomyces pombe*, were shown to copurify with CoQ_6_ or CoQ_10_, respectively (9, 10). Yeast *coq10* mutants respond to antimycin treatment but not myxothiazol, suggesting that Coq10 may function in the delivery of CoQ_6_ to its proper site in the *bc*_1_ complex (14). Furthermore, photoaffinity labeling experiments using a synthetic photoreactive CoQ probe with the purified *S. pombe* Coq10 polypeptide revealed that Coq10 binds the headgroup of CoQ, at a region located within the hydrophobic tunnel formed by its START domain (15). These studies have led to the hypothesis that Coq10 functions as a CoQ chaperone, directing CoQ from its sites of synthesis to its sites of function at the respiratory complexes (8, 14). A role for Coq10 as a CoQ chaperone in respiratory electron transport is further supported by its binding to both the oxidized and reduced forms of the photoreactive azido-quinone probe (15).

In several fungal species, Coq10 and Coq11 evolved as fusion proteins, suggesting a possible functional relationship between these two polypeptides (16). This hypothesis is supported by the observation that subsequent deletion of *COQ11* ameliorates many of the defects of the *coq10*Δ mutant, including restored respiratory growth and CoQ synthome formation (17). These data suggest the Coq11 polypeptide may act as a negative modulator of CoQ biosynthesis and the CoQ synthome (17).

Recent studies have suggested that proper assembly and stability of the yeast CoQ synthome also relies on the presence of the endoplasmic reticulum-mitochondria encounter structure (ERMES) (18, 19) (Fig. 1*B*). ERMES is a multisubunit complex composed of four main proteins (Mmm1, Mdm10, Mdm12, and Mdm34) that tethers the ER to the mitochondria and is essential for biosynthesis and transport of phospholipids between these organelles (20). In addition to its most well-studied role in shuttling phospholipids between the ER and mitochondria, ERMES is proposed to act as a platform for recruiting proteins and other small molecules to the mitochondria based on the needs of the cell (20, 21, 22). Furthermore, yeast lacking individual ERMES constituents (*ERMES*Δ) exhibit distorted mitochondrial morphology, increased loss of mitochondrial DNA, and respiratory deficiency (20, 23, 24, 25). Recently, members of the CoQ synthome were shown to selectively localize into puncta (termed “CoQ domains”) that colocalize with ER-mitochondria contact sites marked by ERMES. Deletion or mutation of individual ERMES subunits results in a loss of CoQ domains, indicating destabilization of the CoQ synthome (18, 19). In accordance with this observation, *ERMES*Δ mutants were found to accumulate steady-state and *de novo* synthesized CoQ_6_ intermediates. These data demonstrate proper CoQ synthome assembly and efficient CoQ_6_ production rely on ERMES complex formation.

In addition to the aforementioned defects attributed to the *coq10*Δ mutant, deletion of *COQ10* results in loss of the spatial relationship between the CoQ domains and ERMES (19). *MDM12*, which encodes the cytosolic subunit of ERMES, is coexpressed with *COQ10 via* a bidirectional promoter, suggesting a functional relationship and/or physical interaction between their gene products (8, 26, 27). While a previous study confirmed that deletion of *MDM12* does not significantly diminish Coq10 protein content (19), it has yet to be determined whether deletion of *COQ10* negatively impacts *MDM12* expression.

In this study, we show that deletion of *COQ10* by replacement of the ORF with the *HIS3* marker (*coq10*Δ) results in diminished Mdm12 protein content. To identify the phenotypes resulting from deletion of the *COQ10* ORF *versus* phenotypes that may be a consequence of disrupted *MDM12* expression and subsequent ERMES dysfunction, we constructed nonfunctional *coq10* mutants that maintain Mdm12 protein content (*coq10-L96S* and *coq10-R147∗*) through the introduction of chromosomal mutations in the *COQ10* ORF. While strains expressing either of the functionally impaired *coq10* mutants phenocopied the respiratory growth defect of the *coq10*Δ mutant, we found that the *coq10-R147∗* mutant, which encodes an unstable truncated Coq10 isoform, maintained a stable CoQ synthome and efficient CoQ_6_ biosynthesis. Based on these data, we propose that the destabilized CoQ synthome and resultant defects in *de novo* CoQ_6_ production observed for the *coq10*Δ mutant are the result of disrupted *MDM12* expression, and therefore ERMES dysfunction, rather than from the loss of the Coq10 polypeptide.

## Results

### Mdm12 protein content is diminished in the yeast *coq10Δ* mutant

Steady-state levels of the Coq10 polypeptide were previously ascertained in *ERMES*Δ mutants, including the *mdm12*Δ mutant, and were found to be similar to that of WT control cells, suggesting that deletion of *MDM12* does not disrupt the expression of *COQ10* despite their coexpression from a bidirectional promoter (19). However, relative protein content of each ERMES component in the *coq10*Δ mutant was not investigated, and it remained uncertain if deletion of *COQ10* impacted Mdm12 protein levels. To this end, we quantified the steady-state levels of Mmm1, Mdm10, and Mdm12 in the *coq10*Δ, *coq11*Δ, and *coq10*Δ*coq11*Δ mutants. For this analysis, we used crude mitochondria to retain the endogenous protein tethers, such as ERMES, that may be lost during the preparation of gradient-purified mitochondria.

**Figure 2:**
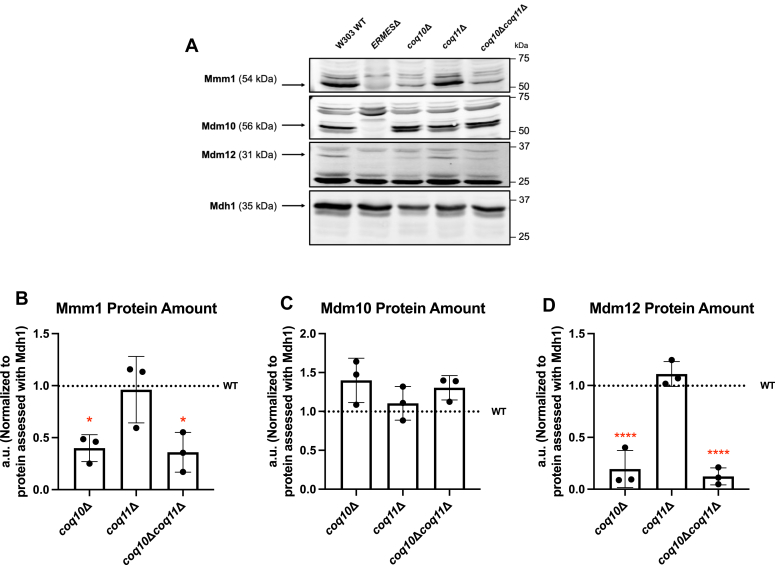
**The Mdm12 and Mmm1 polypeptides are depleted in yeast *coq10*Δ mutants.***A*, aliquots of crude mitochondria (25 μg) from WT, *coq10*Δ, *coq11*Δ, and *coq10*Δ*coq11*Δ yeast strains were subjected to 10% Tris-glycine SDS-PAGE. Immunoblotting was performed with antisera against the indicated ERMES subunits (Mmm1, Mdm10, and Mdm12), and yeast harboring the corresponding deletions were used as negative controls (*ERMES*Δ). Malate dehydrogenase (Mdh1) was used as a loading control. Data are representative of three biological replicates. *B*–*D*, ImageJ was used to quantify triplicate band intensities of select ERMES subunits. Band intensities were normalized to Mdh1 and plotted as percentage of the WT control. The data depict the mean ± SD of three biological replicates. The statistical significance compared with WT is represented by the *red asterisks*; ∗, *p* < 0.05 and ∗∗∗∗, *p* < 0.0001. ERMES, endoplasmic reticulum-mitochondria encounter structure.

Despite the preservation of Coq10 protein content previously observed for *ERMES*Δ mutants, yeast lacking *COQ10* had dramatically reduced amounts of Mdm12 and Mmm1 (Fig. 2). The reduction in Mmm1 protein levels is consistent with previous work that showed the presence of Mdm12 is required for stable expression of Mmm1, and *vice versa* (28). Mdm10 levels were preserved across all mutants, likely due to the involvement of Mdm10 in other mitochondrial import machinery, such as translocase of the outer mitochondrial membrane and sorting and assembly machinery complex biogenesis and function (29). Regardless, the depletion or loss of a single ERMES subunit results in an inability to form the ERMES complex (20), raising concern that some or all of the phenotypes ascribed to the *coq10*Δ mutant could be a consequence of ERMES dysfunction due to diminished *MDM12* expression.

### Chromosomal mutations in the *COQ10* ORF preserve Mdm12 and Mmm1 protein levels

**Figure 3:**
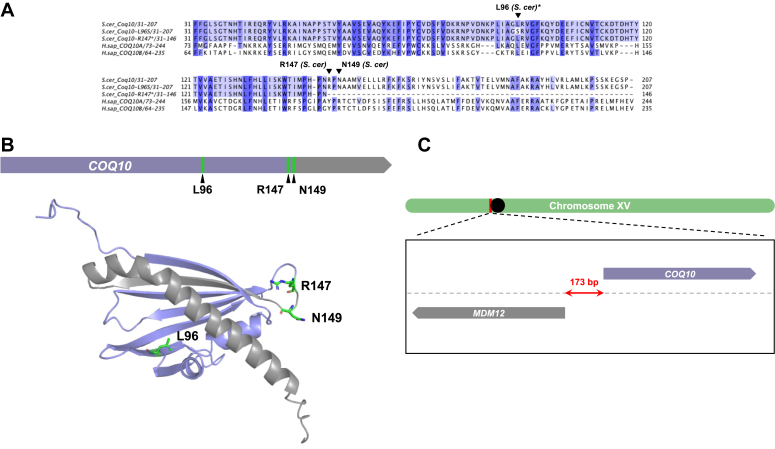
**Structural prediction of *Saccharomyces cerevisiae* Coq10 and multiple sequence alignment with human COQ10 orthologs highlight residues targeted for mutagenesis.***A*, multiple sequence alignment of *S. cerevisiae* Coq10 (residues 31–207) with the Coq10-L96S and Coq10-R147∗ mutant polypeptides constructed in this study and the human homologs COQ10A (residues 73–244)/COQ10B (residues 64–235). The yeast Coq10 polypeptide and orthologous human sequences were obtained from Universal Protein Knowledgebase (UniProtKB). The multiple sequence alignment was constructed using the ClustalW package of Clustal Omega (59) and visualized in JalView2 (60). Conservation of each residue is indicated by degree of shading, which represents 80%, 60% and 40% percent sequence identity from *darkest to lightest shade*, respectively. Residues targeted for mutagenesis in this study are indicated with an *inverted triangle*, and *asterisks* indicate residues deemed critical for ligand binding in previous studies (11). *B*, location of the Coq10 residues targeted for mutagenesis by CRISPR-Cas9 (shown in *green*) within the context of the *COQ10* ORF (*top*) and the AlphaFold predicted structure for *S. cerevisiae* Coq10 (*bottom*, AF-Q08058-F1). The region shown in *gray* represents the truncation that results from introducing the *coq10-R147∗/N149∗* double mutation. *C*, schematic depicting the head-to-head positioning of *COQ10* (*purple*) and *MDM12* (*gray*) within the context of *S. cerevisiae* chromosome XV (*green*). Notably, these two genes are separated by only 173 bps, suggesting deletion of one gene could impact the expression of the other, and *vice versa*.

To distinguish phenotypes that result solely from the absence of the Coq10 polypeptide from those that could be the result of disrupted *MDM12* expression, two separate mutations were introduced into the yeast genome using CRISPR-Cas9 as described in Experimental procedures (30, 31). The first mutation, L96S, is located within the hydrophobic tunnel formed by the Coq10 START domain and is predicted to disrupt ligand binding (Fig. 3, *A* and *B*). Structural and biochemical evidence using Coq10 orthologs from *C. crescentus* (12, 13), *S. pombe* (9), and humans (8) have shown its START domain can directly bind CoQ and its late-stage quinone-containing intermediates. Moreover, expression of the *coq10-L96S* mutant from an integrative locus or a high-copy plasmid in *coq10*Δ yeast fail to rescue respiratory defects, indicating L96 is an important residue for Coq10 function (11). The second mutation, R147∗/N149∗, encodes a truncated isoform of Coq10 where two stop codons were introduced at residues 147 and 149 (hereafter referred to as *coq10-R147∗*) (Fig. 3, *A* and *B*). We rationalized both mutations should be downstream enough from the portion of the *MDM12* promoter region located within the *COQ10* ORF to allow for preservation of *MDM12* expression (Fig. 3*C*).

**Figure 4:**
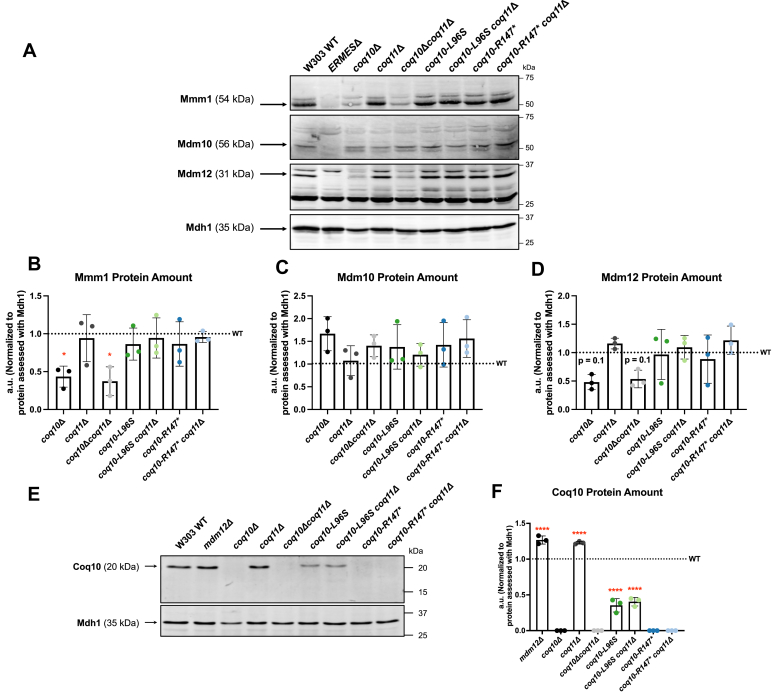
**Mdm12 and Mmm1 protein content is preserved in strains expressing either the Coq10-L96S or Coq10-R147∗ mutant polypeptide.***A*, aliquots of crude mitochondria (25 μg) from the indicated yeast strains were subjected to 10% Tris-glycine SDS-PAGE. Immunoblotting was performed with antisera against the indicated ERMES subunits (Mmm1, Mdm10, and Mdm12), and mitochondria from yeast harboring the corresponding deletions were used as negative controls (*ERMES*Δ). Malate dehydrogenase (Mdh1) was used as a loading control. Data are representative of three biological replicates. *B*–*D*, ImageJ was used to quantify triplicate band intensities of the indicated ERMES proteins. Band intensities were normalized to Mdh1 and plotted as percentage of the WT control. The data depict mean ± SD of three biological replicates, and the statistical significance compared with WT is represented by the *red asterisks* ∗, *p* < 0.05 in panel *B*, or by the stated *p* value in *D*. *E*, 12.5 μg of crude mitochondria were separated on 12% Tris-glycine SDS-PAGE and immunoblotting was performed using Coq10 antisera. An aliquot of mitochondria from the *coq10*Δ yeast was used as a negative control. *F*, ImageJ was used to quantify triplicate band intensities for the Coq10 polypeptide. Band intensities were normalized to Mdh1 and plotted as percentage of the WT control. The data depict the mean ± SD of three biological replicates. The statistical significance compared with WT is represented by the *red asterisks*; ∗∗∗∗, *p* < 0.0001. ERMES, endoplasmic reticulum-mitochondria encounter structure.

Upon successful genomic integration of the *coq10-L96S* and *coq10-R147∗* mutations, we examined the steady-state levels of Coq10 in addition to select ERMES subunits (Mmm1, Mdm10, and Mdm12) (Fig. 4). All ERMES components, including Mdm12, were present at levels similar to that of the WT control in strains harboring either the *coq10-L96S* or *coq10-R147∗* mutation (Fig. 4, *A*–*D*). This observation supports the conclusion that introduction of the selected mutations does not disrupt the promoter region, and therefore expression, of *MDM12*. Additionally, no band corresponding to the Coq10 polypeptide was detected in mitochondria isolated from strains expressing the *coq10-R147∗* mutant (Fig. 4, *E* and *F*).

**Figure 5:**
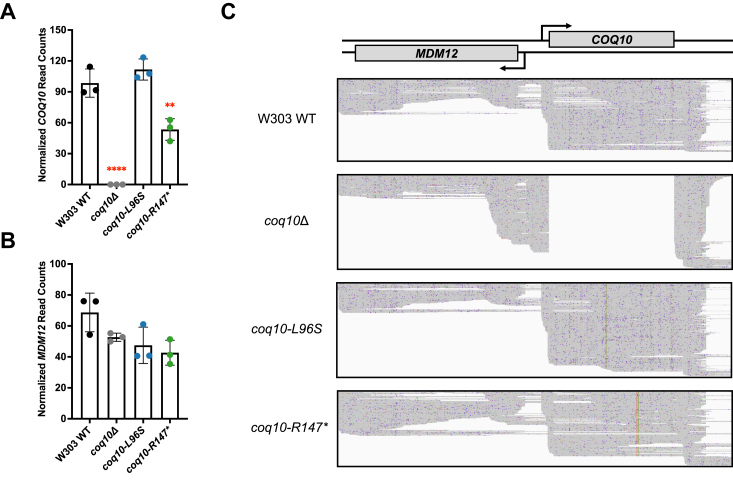
**Insertion of the *HIS3* marker at the *COQ10* locus causes aberrant transcription into the neighboring *MDM12* gene.** The *bar graphs* show normalized read counts for *A*, *COQ10* and *B*, *MDM12* mRNAs based on reads obtained with Oxford Nanopore sequencing using a complementary DNA approach for three replicates per strain (see Experimental procedures). DESeq2 was used to quantify changes in gene expression of the different strains and for normalizing counts to library size. The p-adjusted is used to indicate significance. The data depict the mean ± SD of three biological replicates, and the statistical significance of *COQ10* read counts compared with WT is represented by the *red asterisks*, ∗∗, *p* < 0.01; and ∗∗∗∗, *p* < 0.0001. No significant differences were observed for the *MDM12* read counts. *C*, nanopore sequencing reads detected in the *COQ10* and *MDM12* regions for the indicated strains. Each sequencing read is represented by a horizontal gray line. No reads were detected for the *COQ10* ORF in the *coq10*Δ mutant because of the replacement of the *COQ10* ORF by the *HIS3* marker. Since the reads were aligned to the WT genome, reads corresponding to the *HIS3* gene are not shown. The *HIS3* marker is oriented in a way that positions the *HIS3* promoter adjacent to the 3′ UTR of the deleted *COQ10* ORF.

Given the lack of Coq10 polypeptide in the *coq10-R147∗* mutant, it seemed possible the mRNA produced in the *coq10-R147∗* mutant may be degraded through nonsense-mediated decay due to the presence of the premature stop codons. To address this possibility, we analyzed *COQ10* mRNA content in WT and in the *coq10* mutant strains using Nanopore sequencing as described in Experimental procedures. The use of Nanopore sequencing allowed us to not only quantitate transcript levels, but also to identify and understand any changes in transcript architecture that might have been caused by the introduction of any of the *coq10* mutations described above. As expected, the *coq10*Δ mutation abolished expression of the *COQ10* mRNA (Fig. 5*A*). While the *COQ10* mRNA content in the *coq10-L96S* mutant was not significantly different from that of the WT control, *COQ10* mRNA content was decreased by approximately two-fold in the *coq10-R147∗* mutant compared to WT (Fig. 5*A*). Based on this result, it is unlikely that the absence of the Coq10 polypeptide in the *coq10-R147∗* mutant is solely due to the small quantitative difference in mRNA expression. Rather we speculate that the truncated polypeptide expressed in the *coq10-R147∗* mutant might be unfolded and unstable, which would promote its rapid degradation and lack of detection in this strain.

Our nanopore sequencing approach also allowed us to analyze *MDM12* mRNA content in the *coq10* mutants. In contrast to the changes observed for *COQ10*, *MDM12* mRNA content in each of the *coq10* mutants, including the *coq10*Δ mutant, was not significantly different as compared to the WT control (Fig. 5*B*). However, inspection of the Nanopore sequencing reads revealed that the insertion of the *HIS3* marker cassette used to replace the *COQ10* ORF in the *coq10*Δ mutant resulted in aberrant transcription from the *HIS3* gene into the neighboring *MDM12* gene, producing a large number of transcripts overlapping with the *MDM12* promoter and translation initiation codon, and terminating shortly thereafter (Fig. 5*C*). These *HIS3*-generated transcripts overlapping with the *MDM12* promoter and ORF are likely to repress *MDM12* expression through a combination of transcriptional interference and translational repression, as described recently (32). This interference may explain the decreased production of the Mdm12 polypeptide in the *coq10*Δ strain (Fig. 4*A*). Indeed, such cassette-induced off-target effects have been shown to influence transcription and expression of gene neighbors for select ORFs targeted in the yeast deletion collection (32). In summary, we conclude that the effects observed in the *coq10*Δ mutant on Mdm12 polypeptide content are likely to be due to the repression of *MDM12* through interference from the *HIS3* marker used in the knockout strain. Therefore, using the *coq10-R147∗* mutant provides the best experimental approach for determining which phenotypes are solely due to loss of Coq10 activity, as this mutant maintains protein levels of each ERMES component in the absence of the Coq10 polypeptide.

It is of note that significantly reduced levels of the Coq10-L96S polypeptide were detected when compared with the WT control (Fig. 4, *E* and *F*). The decreased content of the Coq10-L96S polypeptide may confound the assignment of phenotypes as ones that result solely from loss of Coq10 function. However, we decided to proceed with the characterization of both mutant constructs given Mdm12 and Mmm1 are stably expressed in strains harboring either the *coq10-L96S* or *coq10-R147∗* mutation, including those containing a subsequent deletion of *COQ11* (Fig. 4, *A–D*).

### The *coq10* point mutants display impaired respiratory growth similar to the *coq10*Δ mutant

**Figure 6:**
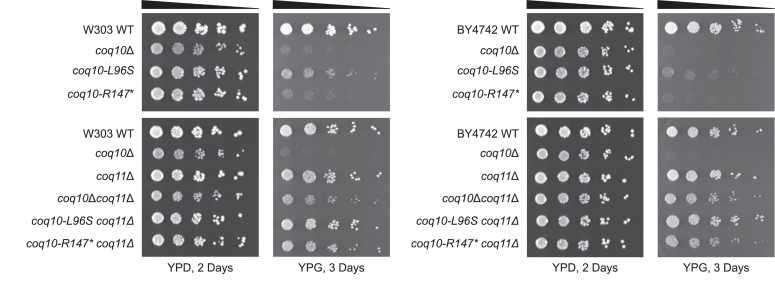
**The *coq10-L96S* and *coq10-R147∗* mutants display impaired respiratory growth similar to the *coq10*Δ mutant that can be restored by deletion of *COQ11*.** Overnight cultures of the indicated yeast strains were diluted to an *A*_600_ = 0.2, and 2 μl of 5-fold serial dilutions were spotted onto fermentable (YPDextrose, YPD) or respiratory (YPGlycerol, YPG) medium. Plates were incubated at 30 °C for 2 or 3 days prior to imaging. Data are representative of three biological replicates.

Given protein levels of ERMES constituents are preserved in strains harboring the *coq10-L96S* or *coq10-R147∗* mutation, we proceeded to reassess phenotypes associated with loss of Coq10 function. The Coq10 polypeptide is required for respiration in yeast (9, 10). As such, yeast *coq10*Δ mutants display impaired growth on medium containing a nonfermentable carbon source, such as yeast extract-peptone-glycerol (YPG). As expected, the *coq10*Δ mutant had poor growth on YPG that was phenocopied by the *coq10-R147∗* mutant across two different genetic backgrounds (Fig. 6). In line with a previous study, the *coq10-L96S* mutant displayed anemic growth on nonfermentable medium that was only slightly improved when compared with the *coq10*Δ and *coq10-R147∗* mutants (11) (Fig. 6). The poor respiratory growth of both the *coq10-R147∗* and *coq10-L96S* mutants indicates the lipid-binding function of Coq10 is necessary for viability on nonfermentable medium, thus supporting the previous conclusion that Coq10 is required for respiration in yeast.

As many defects associated with the deletion of *COQ10* were shown to be alleviated by subsequent deletion of *COQ11* (17), we also sought to reassess this phenotype in *coq11*Δ strains expressing each of the mutant Coq10 isoforms. Like the *coq10*Δ*coq11*Δ mutant, loss of *COQ11* rescued the respiratory growth defect of both the *coq10-R147∗* and *coq10-L96S* mutants in two different yeast genetic backgrounds (Fig. 6). This suggests that deletion of *COQ11* is capable of rescuing the respiratory growth defect of the *coq10*Δ mutant irrespective of the presence of ERMES.

### The *coq10-R147∗* mutant maintains efficient CoQ_6_ biosynthesis

Like most *coq* mutants that display respiratory growth defects, the *coq10*Δ mutant exhibits impaired CoQ_6_ biosynthesis during log phase growth (6, 7, 8, 13). To determine whether this defect in CoQ_6_ biosynthesis can be solely attributed to loss of Coq10, we evaluated *de novo* CoQ_6_ biosynthesis in yeast expressing the mutant Coq10 isoforms by treating yeast cultures of each strain with the isotopically labeled ring precursor, ^13^C_6_-pABA, or ethanol as a vehicle control. Additionally, in accordance with previous work (8, 16, 17), we performed these analyses in both dextrose- and galactose-containing medium to determine carbon source-dependent changes in biosynthetic efficiency. It is important to note only a small percentage (0.2–3%) of CoQ is required for efficient growth on nonfermentable plate medium (5). As such, growth on nonfermentable medium is not always indicative of CoQ biosynthetic efficiency.

**Figure 7:**
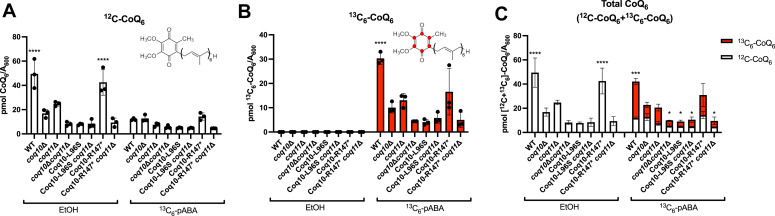
**The *coq10-R147∗* mutant retains the ability to efficiently synthesize CoQ**_**6**_**.** Triplicates of yeast cultured in 25 ml YPGal were labeled at an *A*_600_ ∼0.6 with 8 μg/ml ^13^C_6_-pABA or ethanol as a vehicle control. Fifteen milliliters of each culture were harvested after 5 h, lipid extracted, and analyzed by LC-MS/MS. *A*, unlabeled ^12^C-CoQ_6_; *B*, labeled ^13^C_6_-CoQ_6_; *C*, total amount of CoQ_6_ determined from the sum of ^12^C-CoQ_6_ (*white*) and ^13^C_6_-CoQ_6_ (*red*). The data depict the mean ± SD. The statistical significance as compared with the *coq10*Δ mutant is represented by the *black asterisks*; ∗, *p* < 0.05; ∗∗∗, *p* < 0.001; and ∗∗∗∗, *p* < 0.0001. CoQ, coenzyme Q; pABA, para-aminobenzoic acid; YPGal, yeast extract-peptone-galactose.

**Figure 8:**
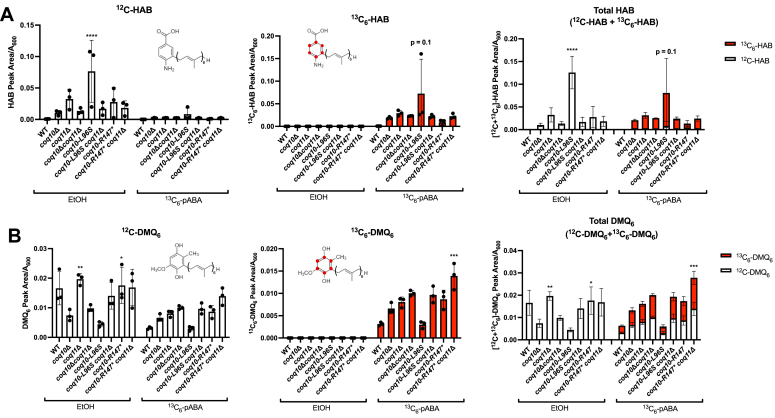
**The *coq10-L96S* mutant has impaired CoQ**_**6**_**biosynthetic efficiency in comparison to *coq10*Δ.** Triplicate of 25 ml cultures in YPGal were labeled at an *A*_600_ ∼0.6 with 8 μg/ml ^13^C_6_-pABA or ethanol. 15 ml of each culture were harvested after 5 h, lipid extracted, and analyzed by LC-MS/MS. *A*, ^12^C-HAB (*white*) and ^13^C_6_-HAB (*red*) and *B*, ^12^C-DMQ_6_ (*white*) and ^13^C_6_-DMQ_6_ (*red*) were measured from whole-cell lipid extracts of the indicated yeast strains. Total HAB and DMQ_6_ were determined from the sum of the respective labeled and unlabeled analytes. The data show mean ± SD. The statistical significance as compared to the *coq10*Δ mutant is represented by the *black asterisks*; ∗, *p* < 0.05; ∗∗, *p* < 0.01; ∗∗∗, *p* < 0.001; and ∗∗∗∗, *p* < 0.0001. CoQ, coenzyme Q; DMQ, demethoxy-Q; HAB, hexaprenylaminobenzoic acid; pABA, para-aminobenzoic acid; YPGal, yeast extract-peptone-galactose.

Consistent with previous studies performed in yeast extract-peptone-galactose (YPGal) medium (17), the *coq10*Δ mutant produced less *de novo* synthesized ^13^C_6_-CoQ_6_ and had decreased total CoQ_6_ content (determined by the sum of ^13^C_6_-CoQ_6_ and unlabeled ^12^C-CoQ_6_) when compared with the W303 WT control (Fig. 7). The *coq10-L96S* mutant produced similar amounts of *de novo* synthesized ^13^C_6_-CoQ_6_ but had decreased total CoQ_6_ content in YPGal when compared with the *coq10*Δ mutant (Fig. 7). Further analyses of key CoQ_6_-intermediates revealed the *coq10-L96S* mutant accumulated the early-stage intermediate ^13^C_6_-hexaprenylaminobenzoic acid (^13^C_6_-HAB) and had decreased amounts of the late-stage intermediate ^13^C_6_-demethoxy-Q_6_ (^13^C_6_-DMQ_6_) when compared with the *coq10*Δ mutant (Fig. 8). These data indicate that the CoQ biosynthetic pathway is less efficient in the *coq10-L96S* mutant when compared to the *coq10*Δ mutant.

In contrast, the *coq10-R147∗* mutant produced elevated amounts of *de novo–*synthesized ^13^C_6_-CoQ_6_ and total CoQ_6_ relative to the *coq10*Δ mutant when cultured in YPGal (Fig. 7). As previously reported (17), changing the carbon source in the medium from galactose to dextrose decreased the CoQ_6_ content across all the *coq10* single mutants, however the *coq10-R147∗* mutant still produced the highest amounts of ^12^C-CoQ_6_ and *de novo–*synthesized ^13^C_6_-CoQ_6_ when compared to the *coq10*Δ mutant (Fig. S1). The elevated CoQ_6_ content and slight decrease in the amount of *de novo–*synthesized ^13^C_6_-HAB as well as total HAB content in the *coq10-R147∗* mutant (Fig. 8) suggests that Coq10 is not required to observe efficient CoQ_6_ biosynthesis.

Mutants lacking both *COQ10* and *COQ11* have decreased total CoQ_6_ content similar to the *coq10*Δ mutant (17). In line with this observation, the *coq10-R147∗* and *coq10-L96S* mutants harboring a subsequent deletion of *COQ11* had decreased amounts of ^12^C-CoQ_6_ and *de novo–*synthesized ^13^C_6_-CoQ_6_ like the *coq10*Δ mutant (Fig. 7, *A* and *B*). Similarly, both double mutants had elevated ^13^C_6_-HAB and ^13^C_6_-DMQ_6_ content, suggesting CoQ biosynthesis is still impaired in the absence of Coq11 (Fig. 8). Taken together, these data support the conclusion that the status of ERMES does not influence the functional relationship between Coq10 and Coq11.

### The *coq10-R147∗* mutant has diminished Coq protein content while still maintaining a stable CoQ synthome

**Figure 9:**
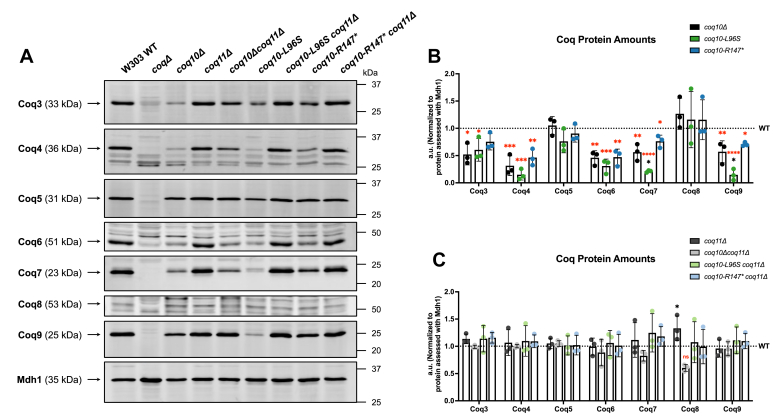
**Decreased abundance of several Coq polypeptides in the *coq10-L96S* and *coq10-R147∗* mutants can be rescued by deletion of *COQ11*.***A*, aliquots of crude mitochondria (12.5 μg) from the indicated yeast strains were subjected to 10% or 12% Tris-glycine SDS-PAGE. Crude mitochondria from *coq3*Δ*-coq9*Δ mutants were included as negative controls for Western blotting using antisera against each of the Coq polypeptides. Mitochondrial malate dehydrogenase (Mdh1) was included as a loading control. Data are representative of three biological replicates. *B*, ImageJ was used to quantify triplicate band intensities for each of the Coq polypeptides. Band intensities were normalized to Mdh1 and plotted as percentage of the WT control. The data depict the mean ± SD of three biological replicates. The statistical significance as compared with WT (*red asterisks*) or the *coq10*Δ mutant (*black asterisks*) are represented by, ∗, *p* < 0.05; ∗∗, *p* < 0.01; ∗∗∗, *p* < 0.001; and ∗∗∗∗, *p* < 0.0001. *C*, blots were quantified as in B. The data depict the mean ± SD of three biological replicates, and the statistical significance as compared with WT (*red asterisks*) or the *coq10*Δ*coq11*Δ mutant (*black asterisks*) are represented by ns, no significance; and ∗, *p* < 0.05.

**Figure 10:**
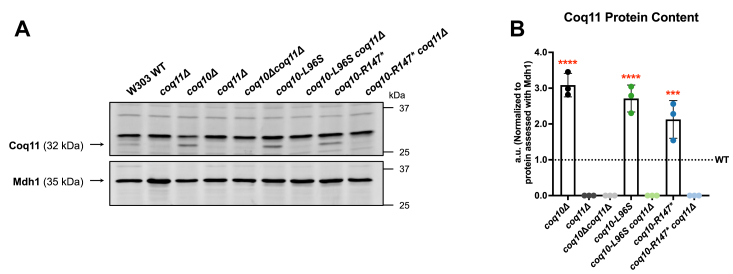
**Relative Coq11 protein abundance is significantly increased in strains harboring *coq10* mutations.***A*, aliquots of crude mitochondria (12.5 μg) from the indicated yeast strains were subjected to 10% Tris-glycine SDS-PAGE. An aliquot of crude mitochondria from the *coq11*Δ mutant was included as a negative control for Western blotting against the Coq11 polypeptide. Mitochondrial malate dehydrogenase (Mdh1) was included as a loading control. The Mdh1 blot is replicated from Fig. 9*A* as the same sets of samples were used across all biological replicates when quantifying protein content for the individual Coq polypeptides. Data are representative of three biological replicates. *B*, ImageJ was used to quantify triplicate band intensities corresponding to Coq11. Band intensities were normalized to Mdh1 and plotted as percentage of the WT control. The data depict the mean ± SD of three biological replicates. The statistical significance compared with WT is represented by the *red asterisks*; ∗, *p* < 0.05; ∗∗, *p* < 0.01; ∗∗∗, *p* < 0.001.

To observe efficient CoQ_6_ biosynthesis in yeast, several Coq polypeptides must assemble into a high-molecular-mass complex termed the CoQ synthome (5). Diminished levels of several Coq polypeptides and a destabilized CoQ synthome are observed in the *coq10*Δ mutant (6, 7). In line with our previous findings, the *coq10-R147∗* mutant phenocopied the *coq10*Δ mutant with respect to having decreased amounts of Coq3, Coq4, Coq6, Coq7, and Coq9 when compared with the WT control (Fig. 9, *A* and *B*). Notably, the *coq10-L96S* mutant contained significantly less Coq7 and Coq9 than the *coq10*Δ mutant (Fig. 9, *A* and *B*). Both the *coq10-R147∗* and *coq10-L96S* mutant displayed elevated Coq11 protein content similar to the *coq10*Δ mutant (Fig. 10).

**Figure 11:**
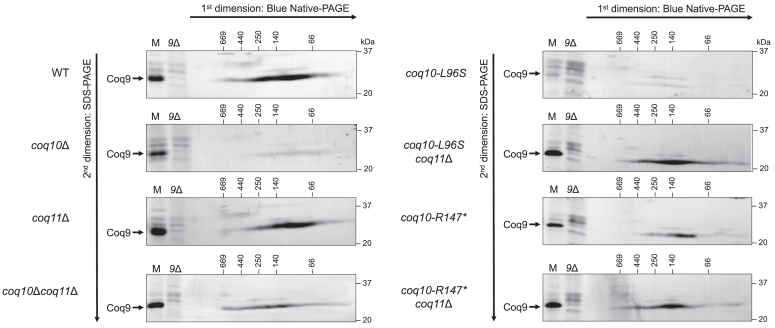
**The *coq10-R147∗* mutant maintains a stable CoQ synthome similar to the WT control.** Aliquots (75 μg) of crude mitochondria isolated from WT, *coq10*Δ, *coq11*Δ, *coq10*Δ*coq11*Δ, *coq10-L96S*, *coq10-L96S coq11*Δ, *coq10-R147∗*, and *coq10-R147∗ coq11*Δ yeast were solubilized with digitonin and separated by 2D BN/SDS-PAGE. Proteins were transferred to polyvinylidene fluoride membranes, and the CoQ synthome was visualized using antisera against Coq9. Aliquots (25 μg) of intact crude mitochondria from each strain (M) and *coq9*Δ (*9*Δ) yeast were included as a loading control and negative control, respectively. BN, blue native; CoQ, coenzyme Q.

While deletion of ERMES subunits does not perturb steady-state levels of the Coq polypeptides, CoQ synthome stability is abolished in the absence of ERMES (19). Given the diminished levels of Mdm12 and Mmm1 in the *coq10*Δ mutant, we assessed CoQ synthome stability by 2D blue native/SDS-PAGE (2D-BN/SDS-PAGE) using Coq9 antisera to indicate complex formation (6) in strains expressing *coq10-R147∗* or *coq10-L96S*. The CoQ synthome in WT yeast can be observed as a large heterogeneous high-molecular-mass complex that spans ∼100 kDa to >1 MDa. The *coq10-L96S* mutant displayed only a very faint signal corresponding to a CoQ synthome (Fig. 11), likely due to its dramatically decreased abundance of several Coq polypeptides as compared with the *coq10*Δ mutant (Fig. 9, *A* and *B*). This suggests that the presence of a nonfunctional Coq10 polypeptide is more detrimental to complex stability than its complete absence. Surprisingly, the mutant expressing the truncated *coq10-R147∗* isoform displayed a stable high-molecular-mass complex similar to the WT control (Fig. 11). This suggests that the preservation of Mdm12 levels in the *coq10-R147∗* mutant allows for proper ERMES formation and, subsequently, maintenance of a stable CoQ synthome. Overall, these observations support a model in which the destabilized CoQ synthome, and resultant inefficient CoQ_6_ biosynthesis, observed in the *coq10*Δ mutant result from loss of ERMES rather than loss of the Coq10 polypeptide.

Subsequent deletion of *COQ11* from the *coq10*Δ mutant has been shown to rescue CoQ synthome formation due to an increased abundance of several Coq polypeptides (17). Like the *coq10*Δ*coq11*Δ mutant, the *coq10-L96S coq11*Δ and *coq10-R147∗ coq11*Δ double mutants displayed restored levels of most Coq polypeptides and a stable CoQ synthome when compared with the WT control (Figs. 9*C* and 11). Notably, the rescued content of Coq polypeptides observed for the *coq10-L96S coq11*Δ double mutant likely explains it’s restored CoQ synthome formation, as indicated by the reappearance of a high-molecular-weight signal in the *coq10-L96S coq11*Δ mutant (Fig. 11).

## Discussion

This work sought to re-evaluate phenotypes attributed to deletion of *COQ10* through introduction of chromosomal mutations within the *COQ10* ORF in *S. cerevisiae*. Previous studies identified *MDM12* as the top-ranked gene coexpressed with *COQ10* in *S. cerevisiae* (8, 26, 27) due to their head-to-head positioning within the yeast genome (Fig. 3). *MDM12* encodes the cytosolic component of the ERMES complex, which acts as a bridge connecting the ER-residing subunit of ERMES, Mmm1, with its mitochondrial components, Mdm10 and Mdm34 (20). While the *mdm12*Δ mutant displays unperturbed steady-state levels of the Coq10 polypeptide (19), we found that the replacement of the *COQ10* ORF with the *HIS3* marker cassette results in a significant decrease in Mdm12 protein content (Fig. 2). Prior work has shown deletion of *MDM12*, and consequently loss of ERMES complex formation, causes decreased respiration (20, 33) and inefficient CoQ_6_ biosynthesis as a result of a destabilized CoQ synthome (19). Given these deleterious phenotypes are shared with yeast harboring a deletion of *COQ10* (10, 13), we sought to distinguish which of the *coq10*Δ phenotypes result solely from loss of the Coq10 polypeptide as opposed to those caused by ERMES defects. Using CRISPR-Cas9 genome editing, we introduced mutations within the *COQ10* ORF that resulted in loss of Coq10 function (*coq10-L96S*) or loss of the Coq10 polypeptide (*coq10-R147∗*) while still preserving Mdm12 protein content (Fig. 4*D*). Our characterization of these mutants demonstrates that while the Coq10 polypeptide is required for respiration (Fig. 6), it is not essential for efficient CoQ_6_ biosynthesis or formation of the CoQ synthome (Figs. 7 and 11).

The function of Coq10 is widely conserved across several organisms, including *C. crescentus* (12, 13), *S. pombe* (9, 15), and humans (8). Notably, the NMR structure of a Coq10 ortholog from *C. crescentus*, CC1736, revealed the presence of a START domain capable of binding CoQ with variable polyisoprenoid chain lengths and its late-stage quinone intermediates (12, 13). Additionally, Coq10 polypeptides from *S. cerevisiae* and *S. pombe* were found to copurify with CoQ_6_ and CoQ_10_, respectively, leading to the hypothesis that Coq10 functions as a CoQ chaperone (9, 10). This hypothesis is further supported by studies showing yeast Coq10 and its orthologs are required for respiration and efficient *de novo* CoQ biosynthesis (8, 14). Surprisingly, the introduction of the L96S mutation within the START domain resulted in more pronounced defects in CoQ_6_ biosynthetic efficiency, Coq polypeptide abundance, and CoQ synthome stability despite the slightly improved respiratory capacity of this mutant when compared to the *coq10*Δ control (Figs. 6, 7, 9, and 11). We posit that the abrogated lipid-binding function of this mutant is compounded by its decreased expression (Fig. 4*E*), resulting in a dominant negative effect. In contrast, the phenotypes displayed by the *coq10-R147∗* mutant manifest solely from loss of the Coq10 polypeptide as opposed to the unforeseen defects that resulted when we expressed the unstable, nonfunctional Coq10-L96S mutant polypeptide. This conclusion is supported by the observation that the *coq10-R147∗* mutant did not exhibit augmented deleterious phenotypes when compared with the *coq10*Δ control.

Stable formation of the CoQ synthome requires the presence of both CoQ and prenylated CoQ-intermediates (7, 34, 35). Studies have shown that bypassing defective steps of the CoQ biosynthetic pathway with appropriate substrate analogs can restore the appearance of CoQ domains, indicating that the late-stage CoQ intermediates are required for CoQ synthome assembly (18, 36). In accordance with these observations, the *coq10*Δ mutant, which produces higher amounts of early-stage intermediates and lower amounts of late-stage intermediates, displays a destabilized CoQ synthome (8, 13, 17). Strikingly, we found that the *coq10-R147∗* mutant, which lacks the Coq10 polypeptide, retained the ability to synthesize CoQ_6_ efficiently and was capable of forming a CoQ synthome similar to that of the WT control (Figs. 7 and 11). Despite its elevated total CoQ_6_ content, the *coq10-R147∗* mutant had defective respiratory growth similar to the *coq10*Δ mutant (Fig. 6). This suggests that the CoQ chaperone function of Coq10 is required mainly for respiration, and is not essential for CoQ biosynthesis and, subsequently, stable formation of the CoQ synthome. We attribute the clear demarcation between respiratory capacity and CoQ synthome stability observed for the *coq10-R147∗* mutant to the preservation of Mdm12 protein content (Fig. 4*D*), thus allowing us to separate phenotypes caused by loss of Coq10 from those that result due to ERMES dysfunction.

Humans possess two distinct Coq10 orthologs, COQ10A and COQ10B (8). Complementation of the yeast *coq10*Δ mutant with either human isoform was shown to rescue defects in respiratory growth on nonfermentable plate medium, supporting the hypothesis that the function of Coq10 as a CoQ chaperone is conserved across organisms. Our data corroborates this conclusion in that both *coq10* mutants constructed in this study display defective respiratory growth on medium containing a nonfermentable carbon source (Fig. 6). In contrast, complementation of the yeast *coq10*Δ mutant with either human Coq10 ortholog failed to fully restore both efficient CoQ_6_ biosynthesis and CoQ synthome formation (8). Our data suggest the negligible effect on CoQ synthome stabilization and CoQ_6_ biosynthesis observed when expressing either COQ10A or COQ10B is a consequence of disrupted ERMES formation in the yeast *coq10*Δ mutant rather than an incompatibility between the human Coq10 orthologs and yeast CoQ biosynthesis.

Coq10 and Coq11 exist as a fusion protein in several *Ustilaginaceae* species, indicating the presence of a functional relationship between these two polypeptides (16). Previous studies have shown that while the *coq11*Δ mutant does not display defects in respiratory growth, it does have decreased *de novo* ^13^C_6_-CoQ_6_ production. Despite its impairment in CoQ_6_ biosynthesis, the *coq11*Δ mutant displays a more stable CoQ synthome as a result of increased Coq polypeptide abundance (16, 17). As such, the Coq11 polypeptide is proposed to play a regulatory role in CoQ_6_ biosynthesis through its function as a negative modulator of CoQ synthome formation (17). This hypothesized function of Coq11 is supported by the observation that deletion of *COQ11* in tandem with *COQ10* rescues the respiratory defect of the *coq10*Δ mutant likely due to increased protein content for several Coq polypeptides and subsequent stabilization of the CoQ synthome (17). Similarly, our *coq10* mutants that harbor a subsequent deletion of *COQ11* displayed restored growth on a nonfermentable medium when compared with the *coq10*Δ mutant (Fig. 6). Furthermore, the *coq10-R147∗ coq11*Δ and *coq10-L96S coq11*Δ double mutants also had decreased *de novo* CoQ_6_ production and restored protein content for several Coq polypeptides like the *coq10*Δ*coq11*Δ mutant (Figs. 7 and 9). This suggests the mechanism by which deletion of *COQ11* rescues the sickly *coq10*Δ phenotypes is independent of ERMES. Elevated Coq11 protein content in all the *coq10* mutants regardless of the type of mutation or its effect on ERMES supports this conclusion (Fig. 10). It is tempting to speculate that the Coq10 polypeptide may negatively regulate Coq11.

A fundamental feature of membrane contact sites is their ability to spatially coordinate cellular processes such that contact sites can serve as nucleation sites for specific pathways depending on the needs of the cell. Previous studies have shown the spatial positioning of the CoQ synthome is mediated by ER-mitochondrial contacts established by ERMES, as evidenced by colocalization of the CoQ domain marker, Coq9-yEGFP, with the ERMES marker, and Mdm34-mCherry (19). Deletion of *COQ10* by its replacement with the *HIS3* marker cassette resulted in significantly less colocalization between the CoQ domains and ERMES (19). Although it was originally hypothesized that Coq10 may modulate the spatial coordination between ERMES and the CoQ domains, our results suggest this is instead the result of disrupted ERMES formation *via* attenuated Mdm12 and Mmm1 protein content in the *coq10*Δ mutant (Fig. 2). While we were able to preserve ERMES protein content in our *coq10* point mutants (Fig. 4, *A*–*D*), it remains unclear if the spatial coordination between ERMES and the CoQ domains is maintained. It is possible that despite the formation of a CoQ synthome in the *coq10-R147∗* mutant (Fig. 11), the Coq10 polypeptide may still be required to mediate the localization of the CoQ synthome alongside ERMES.

Since the initial characterization of the ERMES complex (20), several other ER-mitochondrial contact sites have been discovered (37). The viability of yeast lacking individual ERMES constituents suggests that ERMES-related functions can be compensated for by auxiliary contact sites. In support of this, CoQ domains have been shown to also colocalize with puncta marked by Ltc1 (18), the ER-residing component of an auxiliary ER-mitochondrial tether (38). This suggests it is more important CoQ domains localize to any given ER-mitochondrial contact site rather than contact sites established specifically by ERMES. However, we postulate that there is a unique regulatory relationship between Coq10 and ERMES given the positioning of the *COQ10* and *MDM12* genes within the yeast genome (Fig. 3*C*). Commonly, bidirectional promoters couple two divergent protein-encoding genes involved in related processes to allow for their tight regulation (39, 40). It is possible that coexpression of *COQ10* and *MDM12 via* their shared bidirectional promoter allows for the coupling of CoQ biosynthesis with a transport mechanism capable of distributing CoQ to other cellular membranes. Three ERMES subunits (Mmm1, Mdm12, and Mdm34) possess a synaptotagmin-like mitochondrial lipid binding protein domain shown to facilitate the transport of phospholipids between the ER and mitochondria (20, 21, 33). Structural characterization of ERMES using cryo-correlative light and electron microscopy has shown that these synaptotagmin-like mitochondrial lipid binding protein domains align to form a channel for lipid transport (22).

It is tempting to speculate that ERMES could serve as a platform for the mitochondrial distribution of CoQ in yeast following its synthesis. However, the transport pathway of CoQ from its site of biosynthesis in the inner mitochondrial membrane to the outer membrane and perhaps ERMES is still poorly described. Recent studies identified the Cqd1 and Cqd2 polypeptides to function in the transport of CoQ_6_ to and from the mitochondria, respectively (41). Both Cqd1 and Cqd2 are inner mitochondrial membrane proteins that face the intermembrane space (41), potentially bridging the gap between the CoQ synthome and ERMES. Intriguingly, Cqd1 participates in a contact site between the outer and inner mitochondrial membranes, and overexpression of Cqd1 and Cqd2 elicits contact sites between the ER and mitochondria (42). Taken together, these studies suggest that membrane contact sites likely play significant roles in the trafficking of yeast CoQ_6_ (43).

Despite the absence of a direct human homolog for ERMES, the prevalence of ER-mitochondrial contact sites in humans indicates that spatial regulation of mitochondrial processes *via* contact sites is conserved. For example, knockdown of the gene encoding the mitofusin (MFN) 2 ortholog, *Mfn2*, in mice results in CoQ deficiency and decreased respiratory capacity (44). MFN2 is most commonly known for its role in mitochondrial fusion, but its dual localization to ER and outer mitochondrial membranes enables it to form a physical tether between the two organelles *via* its homodimerization (45). In contrast, MFN1 localizes solely to outer mitochondrial membranes where it can form a heterodimeric tether with ER-localized MFN2 (40, 46). Notably, mice with knockdown of *Mfn1* retain the ability to synthesize CoQ (40). This suggests the CoQ deficiency observed in *Mfn2* knockdown mice is not due to loss of MFN2 function in mitochondrial fusion, but rather results from the loss of MFN2-mediated tethering between the ER and mitochondria. Our work reinforces the finding that ER-mitochondrial contacts can specifically regulate CoQ biosynthesis in that the preservation of ERMES in the *coq10-R147∗* mutant results in *de novo* CoQ production similar to the WT control (Fig. 7).

In summary, this work revealed that previously reported phenotypes attributed to deletion of *COQ10* in *S. cerevisiae* were conflated with those caused by significantly attenuated Mdm12 protein content in the *coq10*Δ mutant. To disentangle these phenotypes, we generated two separate *coq10* point mutants using CRISPR-Cas9 genome editing that maintain Mdm12 protein content and ERMES complex formation. Through our characterization of these mutants, we demonstrate that Coq10 is required for respiration but not efficient CoQ_6_ biosynthesis or formation of the CoQ synthome. Taken together, these results support a model in which Coq10 functions mainly as a CoQ chaperone responsible for directing CoQ from its sites of synthesis to its sites of function at the respiratory complexes.

## Experimental procedures

All reagents were obtained commercially from Thermo Fisher Scientific, unless specified otherwise.

### Yeast strains and growth medium

*Saccharomyces cerevisiae* strains were derived from W303 (47) or S288C (48). Yeast strains used in this study are listed in Table 1. In the S288C genetic background, the complete *COQ10* ORF was replaced with the *KanMX4* selection marker and was generated by the *Saccharomyces* genome KO consortium (see Table 1). In the W303 genetic background, the *COQ10* ORF was replaced with the *HIS3* gene (Table 1). In the latter case, the *COQ10* gene deletion extends from −4 to +6 bp 3′ of the *COQ10* TGA stop codon, corresponding to a deletion of sequence from 310308 to 310941 of chromosome XV.

**Table 1:** Genotype and source of yeast strains

Strain	Genotype	Source
W303 1B	MAT α *leu2-3,-112; his3-11,-15; trp1-1; ura3-1; ade2-1; can1-100*	R. Rothstein^a^
BY4742	MAT α *his3Δ0 leu2Δ0 met15Δ0 ura3Δ0*	(48)
JM6	*MAT a his-4 ρ0*	J. E. McEwen^b^
JM8	*MAT α ade-1 ρ0*	J. E. McEwen^b^
W303a *coq2*Δ	*MAT a, ade2-1 can1-100 his3-11,15 leu2-3,112 trp1-1 ura3-1 coq2::HIS3*	(61)
W303 1B *coq3*Δ	MAT α *leu2-3,-112; his3-11,-15; trp1-1; ura3-1; ade2-1; can1-100 coq3::LEU2*	(62)
W303a *coq4*Δ	MAT a *leu2-3,-112; his3-11,-15; trp1-1; ura3-1; ade2-1; can1-100 coq4::TRP1*	(63)
W303 1B *coq5*Δ	MAT α *leu2-3,-112; his3-11,-15; trp1-1; ura3-1; ade2-1; can1-100 coq5::HIS3*	(64)
W303a *coq6*Δ	MAT a *leu2-3,-112; his3-11,-15; trp1-1; ura3-1; ade2-1; can1-100 coq6::LEU2*	(65)
W303 1B *coq7*Δ	MAT α *leu2-3,-112; his3-11,-15; trp1-1; ura3-1; ade2-1; can1-100 coq7::LEU2*	(66)
W303a *coq8*Δ	MAT a *leu2-3,-112; his3-11,-15; trp1-1; ura3-1; ade2-1; can1-100 coq8::HIS3*	(63)
W303 1B *coq9*Δ	MAT α *leu2-3,-112; his3-11,-15; trp1-1; ura3-1; ade2-1; can1-100 coq9::URA3*	(67)
W303a *coq10*Δ	MAT a *leu2-3,-112; his3-11,-15; trp1-1; ura3-1; ade2-1; can1-100 coq10::HIS3*	(10)
W303 1B *coq11*Δ	MAT α *leu2-3,-112; his3-11,-15; trp1-1; ura3-1; ade2-1; can1-100 coq11::LEU2*	(17)
W303 1B *coq10*Δ*coq11*Δ	MAT α *leu2-3,-112; his3-11,-15; trp1-1; ura3-1; ade2-1; can1-100 coq10::HIS3 coq11::LEU2*	(17)
W303a *mmm1*Δ	MAT a *leu2-3,-112; his3-11,-15; trp1-1; ura3-1; ade2-1; can1-100 mmm1::KanMX*	(33)
W303a *mdm10*Δ	MAT a *leu2-3,-112; his3-11,-15; trp1-1; ura3-1; ade2-1; can1-100 mdm10::HIS3*	(33)
W303a *mdm12*Δ	MAT a *leu2-3,-112; his3-11,-15; trp1-1; ura3-1; ade2-1; can1-100 mdm12::HIS3*	(33)
W303 1B *coq10-L96S*	MAT α *leu2-3,-112; his3-11,-15; trp1-1; ura3-1; ade2-1; can1-100 coq10-L96S*	This work
W303 1B *coq10-L96S coq11*Δ	MAT α *leu2-3,-112; his3-11,-15; trp1-1; ura3-1; ade2-1; can1-100 coq10-L96S coq11::LEU2*	This work
W303 1B *coq10-R147∗*	MAT α *leu2-3,-112; his3-11,-15; trp1-1; ura3-1; ade2-1; can1-100 coq10-R147∗*	This work
W303 1B *coq10-R147∗ coq11*Δ	MAT α *leu2-3,-112; his3-11,-15; trp1-1; ura3-1; ade2-1; can1-100 coq10-R147∗ coq11::LEU2*	This work
BY4741 *coq2*Δ	MAT a *his3Δ0 leu2Δ0 met15Δ0 ura3Δ0 coq2::KanMX4*	(68)
BY4742 *coq10*Δ	MAT α *his3Δ0 leu2Δ0 met15Δ0 ura3Δ0 coq10::KanMX4*	(68)
BY4742 *coq11*Δ	MAT α *his3Δ0 leu2Δ0 met15Δ0 ura3Δ0 coq11::LEU2*	(17)
BY4742 *coq10*Δ*coq11*Δ	MAT α *his3Δ0 leu2Δ0 met15Δ0 ura3Δ0 coq10::HIS3 coq11::LEU2*	(17)
BY4742 *coq10-L96S*	MAT α *his3Δ0 leu2Δ0 met15Δ0 ura3Δ0 coq10-L96S*	This work
BY4742 *coq10-L96S coq11*Δ	MAT α *his3Δ0 leu2Δ0 met15Δ0 ura3Δ0 coq10-L96S coq11::LEU2*	This work
BY4742 *coq10-R147∗*	MAT α *his3Δ0 leu2Δ0 met15Δ0 ura3Δ0 coq10-R147∗*	This work
BY4742 *coq10-R147∗ coq11*Δ	MAT α *his3Δ0 leu2Δ0 met15Δ0 ura3Δ0 coq10-R147∗ coq11::LEU2*	This work

CoQ, coenzyme Q.

a Dr. Rodney Rothstein, Department of Human Genetics, Columbia University.

b Dr. Joan E. McEwen.

Growth media were prepared as described (49) and included YPD (1% yeast extract, 2% peptone, 2% dextrose), YPG (1% yeast extract, 2% peptone, 3% glycerol), and YPGal (1% yeast extract, 2% peptone, 2% galactose, 0.1% dextrose). Plate medium contained 2% bacto-agar.

### Introduction of guide sequences into pCAS by PCR

Guide sequences for introduction of *coq10* point mutations *via* CRISPR-Cas9 genome editing were cloned into the pCAS single-guide RNA cassette as previously described (30, 31). Briefly, point mutations within 20 bp of a Protospace Adjacent Motif (PAM) site and that were located at least halfway in the *COQ10* ORF to avoid disruption of the endogenous *MDM12* promoter were selected for mutagenesis. Guide efficiency was evaluated using the CRISPR design tool in Benchling (Benchling, Inc). Primers for introduction of guide sequences into the pCAS plasmid (Addgene, plasmid #60847) *via* PCR were designed according to Armaleo *et al.* (31), including one mutagenic primer per guide sequence containing the 20-bp guide sequence flanked on either side by 20-bp sequences homologous to the parental pCAS plasmid and a short 20-bp primer whose 5′ end is immediately adjacent to the 5′ end of the mutagenic primer. All primers were 5′ phosphorylated according to standard protocols prior to use. PCR reactions for cloning the guide sequences into the pCAS plasmid were carried out according to the modified protocol described by Armaleo *et al.* (31) and the linear PCR products were blunt-end-ligated using a Quick Ligation Kit (New England Biolabs). The resultant plasmids were transformed into competent *Escherichia coli* DH5α cells (New England Biolabs) and plated on LB + kanamycin (50 μg/ml) medium. Plasmids were isolated from transformants, and correct integration of the guide sequence was verified by Sanger sequencing (Table 2). All primers utilized in pCAS plasmid construction are listed in Table S1.

**Table 2:** Plasmids used in this study

Plasmid	Construct description	Source
pCAS	Expresses *Streptococcus pyogenes* Cas9 plus an HDV ribozyme-sgRNA for genome editing in yeast	(30)
pCAS_C10-L96	pCAS backbone with modified sgRNA-targeting L96 of yeast Coq10	This work
pCAS_C10-R147	pCAS backbone with modified sgRNA-targeting R147 of yeast Coq10	This work

CoQ, coenzyme Q; HDV, hepatitis delta virus; sgRNA, single-guide RNA.

### Use of CRISPR-Cas9 to generate *coq10-L96S* and *coq10-R147∗*

The *coq10* point mutations were introduced chromosomally using CRISPR-Cas9 as previously described (30, 31). Briefly, complementary 60-mer oligonucleotides containing the desired point mutation were designed to serve as the dsDNA repair template (Table S1). The previously designed pCAS plasmids and their corresponding donor oligonucleotides were cotransformed into competent W303 1B or W303 *coq11*Δ yeast cells according to standard yeast transformation protocols (50). Competent yeast cells were prepared according to Ryan *et al.* (30). For each cotransformation, 90 μl competent cells were combined with 1.0 μg pCAS plasmid and 5.0 μg of each complementary donor oligonucleotide. Transformants were selected on YPD + G148 (0.2 mg/ml) plates incubated at 37 °C for three days. Successful transformants were isolated on fresh YPD + G418 plates incubated at 30 °C for two days. Isolated strains were cultured in 5 ml YPD without G418 for 22 h to confer loss of the pCAS plasmid. Cultures were subsequently plated for single colonies onto YPD medium. To confirm the desired mutations, genomic DNA was extracted using the Promega Wizard Genomic DNA Purification Kit (Promega) and the *COQ10* ORF was amplified by PCR and verified by Sanger sequencing (Tables 1 and S1).

### Drop dilution plate assays

Yeast strains were grown overnight in 5 ml of YPD. The following day, cultures were diluted to an *A*_600_ = 0.2 with sterile PBS and 2 μl of 5-fold serial dilutions were spotted onto YPD and YPG plate medium, corresponding to a final *A*_600_ = 0.2, 0.04, 0.008, 0.0016, and 0.00032. Plates were incubated at 30 °C and pictures were taken after two or three days.

### Analysis of *de novo* CoQ and CoQ-intermediates by stable isotope labeling

Cells were grown overnight in 5 ml of YPGal at 30 °C with shaking. The pre-cultures were then back diluted to an *A*_600_ ∼ 0.1 in 25 ml of fresh YPGal and allowed to further expand to midlog phase (*A*_600_ ∼ 0.6). For analysis of *de novo* CoQ biosynthesis, cultures were treated with 8 μg/ml of ^13^C_6_-pABA (Sigma-Aldrich) or ethanol as a vehicle control for 5 h. All cultures were harvested by centrifugation and cell pellets were stored at −20 °C until use.

To prepare for lipid extraction, cell pellets were resuspended in PBS. An aliquot of each cell suspension was added to 2 ml of methanol and cells were lysed by vortexing in the presence of glass beads. Lipids were extracted twice in the presence of the internal standard CoQ_4_ (Sigma-Aldrich) with the addition of 2 ml petroleum ether followed by vortexing each time. A standard curve was constructed by preparing and extracting standards with known amounts of CoQ_6_ (Avanti Polar Lipids) and the internal standard CoQ_4_ alongside the experimental samples. Extracted lipids were dried under N_2_ gas and reconstituted prior to analysis.

Lipid content was analyzed by LC-MS/MS as previously described (8). Briefly, lipids were reconstituted in 200 μl of 1 mg/ml benzoquinone prepared in ethanol and 20 μl of each sample was injected into an API4000 linear tandem mass spectrometer (Applied Biosystems). The instrument’s corresponding analysis software, Analyst version 1.4.2 (https://sciex.com/products/software/analyst-software), was used for data acquisition and processing. CoQ_6_ content was determined by normalizing the peak using the aforementioned standard curve. Relative levels of CoQ-intermediates are represented as peak areas normalized to the internal standard. A one-way ANOVA with multiple comparisons corrected for using Dunnett’s test was performed using GraphPad Prism 10 (https://www.graphpad.com).

### Isolation of crude mitochondria

Yeast strains were cultured overnight in 30 or 50 ml of YPD at 30 °C with shaking. Precultures were back diluted with YPGal and grown with shaking (30 °C, 250 rpm) until cell density reached an *A*_600_ ∼ 4. Spheroplasts were prepared with Zymolyase-20T (MP Biomedicals), and subsequent fractionation steps were carried out as previously described (51). Briefly, spheroplasts were lysed using dounce homogenization and the resulting homogenate was subjected to centrifugation at 1500*g* to pellet large cellular debris and membranes. The supernatant was collected and centrifuged at 12,000*g* to pellet mitochondria. The mitochondrial pellet was washed and centrifuged again at 1500*g* to remove unwanted impurities. The final centrifugation step was conducted at 12,500*g*, and the resultant crude mitochondrial pellet was resuspended in Hepes sorbitol buffer (0.6 M sorbitol, 20 mM Hepes, pH 7.4). Aliquots of crude mitochondria were flash-frozen in liquid nitrogen and stored at −80 °C until further use. All fractionation steps were completed in the presence of EDTA-free protease inhibitor cocktail tablets (Roche), phosphatase inhibitor cocktail set I (Sigma-Aldrich), phosphatase inhibitor cocktail set II (Sigma-Aldrich), and PMSF (Thermo Fisher Scientific), and all centrifugations were conducted at 4 °C. Protein concentration of crude extracts was determined by the bicinchoninic acid assay (Thermo Fisher Scientific). Mitochondria from yeast *ERMES*Δ mutants were prepared in the same manner with the exception that cultures were expanded in YPG to ensure retention of mitochondrial DNA.

### SDS-PAGE and immunoblot analysis of steady-state protein expression

Crude mitochondria (12.5 or 25 μg) were resuspended in SDS sample buffer (50 mM Tris, pH 6.8, 10% glycerol, 2% SDS, 0.1% bromophenol blue, and 1.33% β-mercaptoethanol) and separated by gel electrophoresis on 10 or 12% Tris-glycine polyacrylamide gels. Proteins were transferred to 0.45 μm polyvinylidene fluoride membranes (Millipore) and blocked with blocking buffer (5% milk and 0.1% Tween-20 in phosphate-buffered saline). CoQ polypeptides, ERMES subunits, and mitochondrial protein loading control Mdh1 were detected using rabbit polyclonal antibodies prepared in 0.5% bovine serum albumin or 5% milk at the dilutions listed in Table 3. IRDye 680LT IgG secondary antibodies (LiCOR) were used at a dilution of 1:20,000. Proteins were visualized using the LiCOR Odyssey Infrared Scanner (LiCOR), and immunoblots were quantified by hand using ImageJ software (https://imagej.net; National Institutes of Health, Bethesda, MD). A one-way analysis of variance with multiple comparisons corrected for using Dunnett’s test was performed using GraphPad Prism 10 was used to compare band intensities.

**Table 3:** Description and source of antibodies

Antibody	Working dilution	Source
Coq3	1:200	(69)
Coq4	1:2000	(70)
Coq5	1:5000	(71)
Coq6	1:200	(65)
Coq7	1:500	(72)
Coq8	Affinity-purified, 1:30	(6)
Coq9	1:1000	(6)
Coq10	Affinity-purified, 1:400	(52)
Coq11	1:500	(54)
Mdh1	1:10,000	Lee McAlister-Henn^a^
Mdm10	1:250	(29)
Mdm12	1:200	(29)
Mmm1	1:500	(29)

CoQ, coenzyme Q; Mdh1, mitochondrial malate dehydrogenase.

a Dr. Lee McAlister-Henn, Department of Molecular Biophysics and Biochemistry, University of Texas Health Sciences Center.

### Generation of antibodies against yeast Coq10 and Coq11

To generate antibodies against Coq10, a 0.594 kb fragment of DNA containing the mature *COQ10* ORF (F_31_ → P_207_) was amplified from genomic yeast DNA by PCR with the forward primer Nde1_tCoq10_FP (5′ GGCCCATATGTTTTTTGGTTTGAGCGG 3′), encoding an initiator Met codon in frame to the mature F_31_ codon of the *COQ10* ORF and adjacent coding sequence (+91 to +107). The reverse primer, BamH1_tCoq10_RP (5′GGCCGGATCCTCAGTGATGATGGTGATGGTGACCACTAGCAGAACCGGAACCCTGAAAATAGAGATTTTCCCCCGGAGAGCCTTCTTT 3′), encodes five carboxy-terminal residues of Coq10, (bold underline designates +207 to +193 of the *COQ10* ORF). The interceding sequence corresponds to a tobacco etch virus protease site (dotted underline), linkers, and the 6 × His-tag (dashed underline). The amplified DNA was digested with *Nde*1 (underlined) and *Bam*H1 (double underlined). The digested DNA fragment was ligated into the *Nde*1 and *Bam*H1 sites of the expression vector pET-15b. The resulting plasmid pET15b_tCoq10, expressed yeast Coq10 with a 6 × His-tag at its C terminus. The resulting 23 kDa fusion protein was overexpressed in the *E. coli* BL21 (DE3) strain (New England Biolabs). Cell pellets were harvested by centrifugation and approximately 30 g of cells were resuspended in lysis buffer (50 mM NaH_2_PO_4_, 300 mM NaCl, 10 mM imidazole pH 8.0, 5 mM β-mercaptoethanol, 5% glycerol, and 0.4% dodecyl maltoside). One tablet of Roche EDTA-free protease inhibitor was added per 80 ml lysis buffer and the cell density was approximately 37% weight/volume. Cells were disrupted with a microfluidizer and the cell lysate was clarified by centrifugation at 12,000*g* for 5 min at 4 °C. The fusion protein in the resultant supernatant was then purified with Ni-NTA Superflow resin (Qiagen) as previously described (52). The eluted protein was concentrated with a 10 kDa Amicon filter, and then subjected to size-exclusion chromatography using a Superdex 200 10/300 GL column. The purified Coq10-His_6_-tagged protein was used to raise polyclonal antisera in rabbits by a standard immunization protocol (Cocalico Biologicals, Inc). The specificity of the polyclonal antisera was determined by immunoblotting with crude or gradient-purified mitochondria from WT and *coq10*Δ mutant yeast (52). When necessary, the antisera were affinity purified as described (53).

To generate antibodies against Coq11, a synthetic peptide antigen specific to the *COQ11* ORF (K_116_-F_130_; KKSKKEQEKANQRSF) was conjugated to keyhole limpet hemocyanin and used to elicit an immune response in rabbits by standard protocols (Cocalico Biologicals).

The specificities of the polyclonal antisera were determined by immunoblotting with crude or gradient-purified mitochondria from WT and *coq11*Δ null mutant yeast (54).

### RNA isolation

Overnight cultures of WT (W303 1B), W303a *coq10*Δ, W303 1B *coq10-L96S*, and W303 1B *coq10-R147∗* yeast in YPGal medium were back-diluted with 30 ml fresh YPGal medium to *A*_600_ = 0.2, and grown to *A*_600_ = 1 (log phase). Cells were harvested by centrifugation at 1950*g* for 5 min, washed with 10 ml sterile water, and transferred to 2 ml screw-cap tubes. Cell pellets were flash-frozen in liquid nitrogen and stored at −80 °C until RNA extractions were carried out.

To frozen cell pellets, 500 μl of phenol-chloroform (phenol: chloroform: isoamyl alcohol 25:24:1, pH 8.0, Thermo Fisher Scientific), 500 μl of RNA-SDS buffer (50 mM Tris–HCl, pH 7.5, 100 mM NaCl, 10 mM EDTA, and 2% SDS w/v), and acid-washed glass beads were added and vortexed for 1 min. Samples were heated at 65 °C for 6 min, vortexed for another min, and subjected to centrifugation at 12,000*g* for 5 min to allow phase separation. The top aqueous layer was transferred to a new Eppendorf tube with 450 μl of fresh phenol-chloroform before vortexing and centrifugation as before. The top aqueous layer was transferred again to a new tube with 1 ml of ethanol and 40 μl of 3 M sodium acetate, pH 5.2 and cooled to −80 °C to facilitate RNA precipitation. Samples were subjected to centrifugation (12,000*g*), and the resulting RNA pellets were washed with 70% ethanol and treated with DNase I (New England Biolabs) before the final resuspension in nuclease-free water.

### Nanopore sequencing and analyses of mRNA content

RNA libraries were prepared from 500 ng of total RNAs using the PCR-complementary DNA barcoding kit from Oxford Nanopore (ONT, catalog #: SQK-PCB114.24) as per the manufacturer's instructions. Sequencing was performed using R10.4.1 flow cells on a MinION Mk1B device and sequenced for 48 h. Base calling was performed using the built in Dorado basecaller in Minknow (Dorado version: 7.4.12; https://github.com/nanoporetech/dorado/releases). Reads were then mapped to the *Saccharomyces cerevisiae* genome (S288C_reference_sequence_R64-3-1) using Minimap 2 (Version 2.17-r941; https://github.com/lh3/minimap2). Reads were visualized using IGV (Version 2.12.3; https://igv.org). For read counts we calculated the number of reads mapping to each gene using featureCounts (55). DESeq2 (https://bioconductor.org) was used to quantify changes in gene expression of the different strains and for normalizing counts to library size (56).

### 2D BN/SDS-PAGE of high-molecular-mass complexes

2D-BN/SDS-PAGE was performed as described previously (57, 58). Crude mitochondria (300 μg) were solubilized for 1 h on ice with 16 mg/ml digitonin (Biosynth) in the presence of the protease and phosphatase inhibitors used during mitochondrial isolation. Solubilized protein was quantified using the bicinchoninic acid assay. Seventy-five micrograms of solubilized mitochondria were separated on Native-PAGE 4 to 16% Bis-Tris gels (Invitrogen) and cut into strips for the second-dimension separation. Gel strips were then further separated on 10% Tris-glycine polyacrylamide gels. Following the second-dimension separation, immunoblot analyses of the CoQ synthome was performed as described above using an antibody against Coq9. Lyophilized protein used for the native gel high molecular weight standards were obtained from GE Healthcare (Sigma-Aldrich).

## Data availability

Nanopore sequencing data have been submitted to the GEO repository, accession number GSE276665, to be made available September 13, 2024. This article contains supporting information. All study data are included in the article and/or supporting information.

## Supporting information

This article contains supporting information.

## Conflict of interest

The authors declare that they have no conflicts of interest with the contents of this article.

## References

[1] Banerjee R., Purhonen J., Kallijarvi J. (2022). The mitochondrial coenzyme Q junction and complex III: biochemistry and pathophysiology. FEBS J..

[2] Guerra R.M., Pagliarini D.J. (2023). Coenzyme Q biochemistry and biosynthesis. Trends Biochem. Sci..

[3] Okada K., Suzuki K., Kamiya Y., Zhu X., Fujisaki S., Nishimura Y. (1996). Polyprenyl diphosphate synthase essentially defines the length of the side chain of ubiquinone. Biochim. Biophys. Acta..

[4] Frei B., Kim M.C., Ames B.N. (1990). Ubiquinol-10 is an effective lipid-soluble antioxidant at physiological concentrations. Proc. Natl. Acad. Sci. U. S. A..

[5] Awad A.M., Bradley M.C., Fernandez-Del-Rio L., Nag A., Tsui H.S., Clarke C.F. (2018). Coenzyme Q10 deficiencies: pathways in yeast and humans. Essays Biochem..

[6] Hsieh E.J., Gin P., Gulmezian M., Tran U.C., Saiki R., Marbois B.N. (2007). *Saccharomyces cerevisiae* Coq9 polypeptide is a subunit of the mitochondrial coenzyme Q biosynthetic complex. Arch. Biochem. Biophys..

[7] He C.H., Xie L.X., Allan C.M., Tran U.C., Clarke C.F. (2014). Coenzyme Q supplementation or over-expression of the yeast Coq8 putative kinase stabilizes multi-subunit Coq polypeptide complexes in yeast *coq* null mutants. Biochim. Biophys. Acta (BBA) - Mol. Cell Biol. Lipids.

[8] Tsui H.S., Pham N.V.B., Amer B.R., Bradley M.C., Gosschalk J.E., Gallagher-Jones M. (2019). Human COQ10A and COQ10B are distinct lipid-binding START domain proteins required for coenzyme Q function. J. Lipid Res..

[9] Cui T.Z., Kawamukai M. (2009). Coq10, a mitochondrial coenzyme Q binding protein, is required for proper respiration in *Schizosaccharomyces pombe*. FEBS J..

[10] Barros M.H., Johnson A., Gin P., Marbois B.N., Clarke C.F., Tzagoloff A. (2005). The *Saccharomyces cerevisiae COQ10* gene encodes a START domain protein required for function of coenzyme Q in respiration. J. Biol. Chem..

[11] Busso C., Bleicher L., Ferreira J.R., Barros M.H. (2010). Site-directed mutagenesis and structural modeling of Coq10p indicate the presence of a tunnel for coenzyme Q_6_ binding. FEBS Lett..

[12] Shen Y., Goldsmith-Fischman S., Atreya H.S., Acton T., Ma L., Xiao R. (2005). NMR structure of the 18 kDa protein CC1736 from *Caulobacter crescentus* identifies a member of the START domain superfamily and suggests residues mediating substrate specificity. Proteins.

[13] Allan C.M., Hill S., Morvaridi S., Saiki R., Johnson J.S., Liau W.S. (2013). A conserved START domain coenzyme Q-binding polypeptide is required for efficient Q biosynthesis, respiratory electron transport, and antioxidant function in *Saccharomyces cerevisiae*. Biochim. Biophys. Acta.

[14] Busso C., Tahara E.B., Ogusucu R., Augusto O., Ferreira-Junior J.R., Tzagoloff A. (2010). *Saccharomyces cerevisiae coq10* null mutants are responsive to antimycin A. FEBS J..

[15] Murai M., Matsunobu K., Kudo S., Ifuku K., Kawamukai M., Miyoshi H. (2014). Identification of the binding site of the quinone-head group in mitochondrial Coq10 by photoaffinity labeling. Biochemistry.

[16] Allan C.M., Awad A.M., Johnson J.S., Shirasaki D.I., Wang C., Blaby-Haas C.E. (2015). Identification of Coq11, a new coenzyme Q biosynthetic protein in the CoQ-Synthome in *Saccharomyces cerevisiae*. J. Biol. Chem..

[17] Bradley M.C., Yang K., Fernandez-Del-Rio L., Ngo J., Ayer A., Tsui H.S. (2020). *COQ11* deletion mitigates respiratory deficiency caused by mutations in the gene encoding the coenzyme Q chaperone protein Coq10. J. Biol. Chem..

[18] Subramanian K., Jochem A., Le Vasseur M., Lewis S., Paulson B.R., Reddy T.R. (2019). Coenzyme Q biosynthetic proteins assemble in a substrate-dependent manner into domains at ER-mitochondria contacts. J. Cell Biol..

[19] Eisenberg-Bord M., Tsui H.S., Antunes D., Fernandez-Del-Rio L., Bradley M.C., Dunn C.D. (2019). The endoplasmic reticulum-mitochondria encounter structure complex coordinates coenzyme Q biosynthesis. Contact (Thousand Oaks).

[20] Kornmann B., Currie E., Collins S.R., Schuldiner M., Nunnari J., Weissman J.S. (2009). An ER-mitochondria tethering complex revealed by a synthetic biology screen. Science.

[21] AhYoung A.P., Jiang J., Zhang J., Khoi Dang X., Loo J.A., Zhou Z.H. (2015). Conserved SMP domains of the ERMES complex bind phospholipids and mediate tether assembly. Proc. Natl. Acad. Sci. U. S. A..

[22] Wozny M.R., Di Luca A., Morado D.R., Picco A., Khaddaj R., Campomanes P. (2023). In situ architecture of the ER-mitochondria encounter structure. Nature.

[23] Berger K.H., Sogo L.F., Yaffe M.P. (1997). Mdm12p, a component required for mitochondrial inheritance that is conserved between budding and fission yeast. J. Cell Biol..

[24] Hobbs A.E., Srinivasan M., McCaffery J.M., Jensen R.E. (2001). Mmm1p, a mitochondrial outer membrane protein, is connected to mitochondrial DNA (mtDNA) nucleoids and required for mtDNA stability. J. Cell Biol..

[25] Youngman M.J., Hobbs A.E., Burgess S.M., Srinivasan M., Jensen R.E. (2004). Mmm2p, a mitochondrial outer membrane protein required for yeast mitochondrial shape and maintenance of mtDNA nucleoids. J. Cell Biol..

[26] Okamura Y., Aoki Y., Obayashi T., Tadaka S., Ito S., Narise T. (2015). COXPRESdb in 2015: coexpression database for animal species by DNA-microarray and RNAseq-based expression data with multiple quality assessment systems. Nucl. Acids Res..

[27] Hibbs M.A., Hess D.C., Myers C.L., Huttenhower C., Li K., Troyanskaya O.G. (2007). Exploring the functional landscape of gene expression: directed search of large microarray compendia. Bioinformatics.

[28] Meisinger C., Pfannschmidt S., Rissler M., Milenkovic D., Becker T., Stojanovski D. (2007). The morphology proteins Mdm12/Mmm1 function in the major beta-barrel assembly pathway of mitochondria. EMBO J..

[29] Ellenrieder L., Opalinski L., Becker L., Kruger V., Mirus O., Straub S.P. (2016). Separating mitochondrial protein assembly and endoplasmic reticulum tethering by selective coupling of Mdm10. Nat. Commun..

[30] Ryan O.W., Poddar S., Cate J.H. (2016). CRISPR-Cas9 genome Engineering in *Saccharomyces cerevisiae* cells. Cold Spring Harb. Protoc..

[31] Armaleo D., Chiou L. (2021). Modeling in yeast how rDNA introns slow growth and increase desiccation tolerance in lichens. G3 (Bethesda).

[32] Powers E.N., Chan C., Doron-Mandel E., Llacsahuanga Allcca L., Kim Kim J., Jovanovic M. (2022). Bidirectional promoter activity from expression cassettes can drive off-target repression of neighboring gene translation. eLife.

[33] Tan T., Ozbalci C., Brugger B., Rapaport D., Dimmer K.S. (2013). Mcp1 and Mcp2, two novel proteins involved in mitochondrial lipid homeostasis. J. Cell Sci..

[34] Tran U.C., Clarke C.F. (2007). Endogenous synthesis of coenzyme Q in eukaryotes. Mitochondrion.

[35] Wang Y., Hekimi S. (2019). The Complexity of making ubiquinone. Trends Endocrinol. Metab..

[36] Ozeir M., Muhlenhoff U., Webert H., Lill R., Fontecave M., Pierrel F. (2011). Coenzyme Q biosynthesis: coq6 is required for the C5-Hydroxylation reaction and substrate analogs rescue Coq6 deficiency. Chem. Biol..

[37] Eisenberg-Bord M., Shai N., Schuldiner M., Bohnert M. (2016). A tether is a tether is a tether: tethering at membrane contact sites. Dev. Cell.

[38] Murley A., Sarsam R.D., Toulmay A., Yamada J., Prinz W.A., Nunnari J. (2015). Ltc1 is an ER-localized sterol transporter and a component of ER-mitochondria and ER-vacuole contacts. J. Cell Biol..

[39] Wei W., Pelechano V., Jarvelin A.I., Steinmetz L.M. (2011). Functional consequences of bidirectional promoters. Trends Genet..

[40] Arnone J.T., Robbins-Pianka A., Arace J.R., Kass-Gergi S., McAlear M.A. (2012). The adjacent positioning of co-regulated gene pairs is widely conserved across eukaryotes. BMC Genomics.

[41] Kemmerer Z.A., Robinson K.P., Schmitz J.M., Manicki M., Paulson B.R., Jochem A. (2021). UbiB proteins regulate cellular CoQ distribution in *Saccharomyces cerevisiae*. Nat. Commun..

[42] Khosravi S., Chelius X., Unger A.K., Rieger D., Frickel J., Sachsenheimer T. (2023). The UbiB family member Cqd1 forms a novel membrane contact site in mitochondria. J. Cell Sci..

[43] Guile M.D., Jain A., Anderson K.A., Clarke C.F. (2023). New insights on the uptake and trafficking of coenzyme Q. Antioxidants (Basel).

[44] Mourier A., Motori E., Brandt T., Lagouge M., Atanassov I., Galinier A. (2015). Mitofusin 2 is required to maintain mitochondrial coenzyme Q levels. J. Cell Biol..

[45] Wilson E.L., Metzakopian E. (2021). ER-mitochondria contact sites in neurodegeneration: genetic screening approaches to investigate novel disease mechanisms. Cell Death Differ..

[46] Zung N., Schuldiner M. (2020). New horizons in mitochondrial contact site research. Biol. Chem..

[47] Thomas B.J., Rothstein R. (1989). Elevated recombination rates in transcriptionally active DNA. Cell.

[48] Brachmann C.B., Davies A., Cost G.J., Caputo E., Li J., Hieter P. (1998). Designer deletion strains derived from *Saccharomyces cerevisiae* S288C: a useful set of strains and plasmids for PCR-mediated gene disruption and other applications. Yeast.

[49] Burke D., Dawson D., Stearns T. (2000). Methods in Yeast Genetics.

[50] Gietz R.D., Woods R.A. (2002). Transformation of yeast by lithium acetate/single-stranded carrier DNA/polyethylene glycol method. Methods Enzymol..

[51] Xie L.X., Hsieh E.J., Watanabe S., Allan C.M., Chen J.Y., Tran U.C. (2011). Expression of the human atypical kinase ADCK3 rescues coenzyme Q biosynthesis and phosphorylation of Coq polypeptides in yeast *coq8* mutants. Biochim. Biophys. Acta.

[52] Tsui H.S. (2019).

[53] Pauli D., Tonka C.H., Tissieres A., Arrigo A.P. (1990). Tissue-specific expression of the heat shock protein HSP27 during Drosophila melanogaster development. J. Cell Biol..

[54] Bradley M.C. (2020).

[55] Liao Y., Smyth G.K., Shi W. (2014). featureCounts: an efficient general purpose program for assigning sequence reads to genomic features. Bioinformatics.

[56] Love M.I., Huber W., Anders S. (2014). Moderated estimation of fold change and dispersion for RNA-seq data with DESeq2. Genome Biol..

[57] Schagger H., Cramer W.A., von Jagow G. (1994). Analysis of molecular masses and oligomeric states of protein complexes by blue native electrophoresis and isolation of membrane protein complexes by two-dimensional native electrophoresis. Anal. Biochem..

[58] Wittig I., Braun H.P., Schagger H. (2006). Blue native PAGE. Nat. Protoc..

[59] Sievers F., Higgins D.G. (2018). Clustal Omega for making accurate alignments of many protein sequences. Protein Sci..

[60] Waterhouse A.M., Procter J.B., Martin D.M., Clamp M., Barton G.J. (2009). Jalview Version 2--a multiple sequence alignment editor and analysis workbench. Bioinformatics.

[61] Ashby M.N., Kutsunai S.Y., Ackerman S., Tzagoloff A., Edwards P.A. (1992). *COQ2* is a candidate for the structural gene encoding para-hydroxybenzoate:polyprenyltransferase. J. Biol. Chem..

[62] Do T.Q., Schultz J.R., Clarke C.F. (1996). Enhanced sensitivity of ubiquinone-deficient mutants of *Saccharomyces cerevisiae* to products of autoxidized polyunsaturated fatty acids. Proc. Natl. Acad. Sci. U. S. A..

[63] Hsu A.Y., Do T.Q., Lee P.T., Clarke C.F. (2000). Genetic evidence for a multi-subunit complex in the O-methyltransferase steps of coenzyme Q biosynthesis. Biochim. Biophys. Acta (BBA) - Mol. Cell Biol. Lipids.

[64] Barkovich R.J., Shtanko A., Shepherd J.A., Lee P.T., Myles D.C., Tzagoloff A. (1997). Characterization of the *COQ5* gene from *Saccharomyces cerevisiae*. Evidence for a C-methyltransferase in ubiquinone biosynthesis. J. Biol. Chem..

[65] Gin P., Hsu A.Y., Rothman S.C., Jonassen T., Lee P.T., Tzagoloff A. (2003). The *Saccharomyces cerevisiae COQ6* gene encodes a mitochondrial flavin-dependent monooxygenase required for coenzyme Q biosynthesis. J. Biol. Chem..

[66] Marbois B.N., Clarke C.F. (1996). The *COQ7* gene encodes a protein in *Saccharomyces cerevisiae* necessary for ubiquinone biosynthesis. J. Biol. Chem..

[67] Johnson A., Gin P., Marbois B.N., Hsieh E.J., Wu M., Barros M.H. (2005). *COQ9*, a new gene required for the biosynthesis of coenzyme Q in *Saccharomyces cerevisiae*. J. Biol. Chem..

[68] Winzeler E.A., Shoemaker D.D., Astromoff A., Liang H., Anderson K., Andre B. (1999). Functional characterization of the *S. cerevisiae* genome by gene deletion and Parallel analysis. Science.

[69] Poon W.W., Barkovich R.J., Hsu A.Y., Frankel A., Lee P.T., Shepherd J.N. (1999). Yeast and rat Coq3 and *Escherichia coli* UbiG polypeptides catalyze both *O*-methyltransferase steps in coenzyme Q biosynthesis. J. Biol. Chem..

[70] Belogrudov G.I., Lee P.T., Jonassen T., Hsu A.Y., Gin P., Clarke C.F. (2001). Yeast *COQ4* encodes a mitochondrial protein required for coenzyme Q synthesis. Arch. Biochem. Biophys..

[71] Baba S.W., Belogrudov G.I., Lee J.C., Lee P.T., Strahan J., Shepherd J.N. (2004). Yeast Coq5 C-methyltransferase is required for stability of other polypeptides involved in coenzyme Q biosynthesis. J. Biol. Chem..

[72] Tran U.C., Marbois B., Gin P., Gulmezian M., Jonassen T., Clarke C.F. (2006). Complementation of *Saccharomyces cerevisiae coq7* mutants by mitochondrial targeting of the *Escherichia coli* UbiF polypeptide. Two functions of yeast Coq7 polypeptide in coenzyme Q biosynthesis. J. Biol. Chem..

