# Supplementary material for: Nonfunctional coq10 mutants maintain the ERMES complex and reveal true phenotypes associated with the loss of the coenzyme Q chaperone protein Coq10
Source: J Biol Chem. 2024 Sep 27;300(11):107820. doi: 10.1016/j.jbc.2024.107820 (PMC11541779; doi:10.1016/j.jbc.2024.107820)
Supplement: Supporting information [file mmc1.docx]

Nonfunctional *coq10* mutants maintain the Endoplasmic Reticulum-Mitochondria Encounter Structure and reveal true phenotypes associated with the loss of the coenzyme Q chaperone protein Coq10

**Noelle Alexa Novales^1‡^, Kelsey J. Feustel^1‡^, Kevin L. He^1^, Guillaume F. Chanfreau^1^, and**

**Catherine F. Clarke^1*^**

**Contents of Supporting Information**

**Table S1**

**Figure S1**

**SUPPORTING INFORMATION**

**Table S1. Primers and oligonucleotides used for CRISPR-Cas9 gene editing**

| Name | Sequence (5ʹ-3ʹ) |
| --- | --- |
| pCas9_Amplify_Rev | AAGGTGTTGCCCAGCCGGCG |
| C10-L96-gRNA-F | CGGGTGGCGAATGGGACTTTCTCATTGCGGGGCTTCGAGTGTTTTAG AGCTAGAAATAGC |
| C10-R147-gRNA-F | CGGGTGGCGAATGGGACTTTTCACCCAAATAGACCAAATGGTTTTA GAGCTAGAAATAGC |
| pCas9_Check | CGGAATAGGAACTTCAAAGCGT |
| C10-L96S-dDNA-F^a^ | CCTGTGGATAACAAGCCTCTCATTGCGGGG**AG**TCGAGTTGGTTTCAA ACAATACGATGAG |
| C10-L96S-dDNA-R^a^ | CTCATCGTATTGTTTGAAACCAACTCGA**CT**CCCCGCAATGAGAGGCT TGTTATCCACAGG |
| C10-R147-dDNA-F^a^ | GAAATGGACCATAATGCCTCACCCAAAT**T**GACCA**TGA**GCGGCCATG GTAGAACTTCTATT |
| C10-R147-dDNA-R^a^ | AATAGAAGTTCTACCATGGCCGC**TCA**TGGTC**A**ATTTGGGTGAGGCA TTATGGTCCATTTC |
| COQ10_Int_Fwd | GGCCCTCACAGACATTGATA |
| COQ10_Int_Rev | TCACGGAGAGCCTTCTTTAG |
| COQ10_KO_Fwd | CCGTTACATAGGATGGCATG |
| COQ10_KO_Rev | CCCCTACTGGAGCTTGTTAG |
| COQ11_KO_Fwd | AGTGTCTCCTCGTAATGCCATC |
| COQ11_KO_Rev | CAACCAAGAGGCATATCAGGC |

^a^ Bases that are bolded and underlined denote bases mutated as compared with the wild-type *COQ10* ORF

**Figure S1.** The *coq10-R147** mutant retains the ability to synthesize CoQ_6_ in dextrose-containing growth medium. Triplicate of 25 mL cultures in YPD were labeled at an *A*_600_ ~ 0.6 with 8 µg/ml ^13^C_6_-pABA or ethanol as a vehicle control. 15 mL of each culture were harvested after 5 hours, lipid extracted, and analyzed by LC-MS/MS. ^12^C-CoQ_6_ and ^13^C_6_-CoQ_6_ are represented in white and red, respectively, and the total CoQ_6_ content is determined by the sum of unlabeled and labeled CoQ_6_. The data show mean ± SD, and the statistical significance as compared to the *coq10*Δ mutant is represented by *black asterisks*; *, *p* < 0.05; **, *p* < 0.01; ***, *p* < 0.001; and ****, *p* < 0.0001.
